# Photon-counting CT for forensic death investigations—a glance into the future of virtual autopsy

**DOI:** 10.3389/fradi.2024.1463236

**Published:** 2024-09-16

**Authors:** Dominic Gascho

**Affiliations:** Department of Forensic Medicine and Imaging, Institute of Forensic Medicine, University of Zurich, Zurich, Switzerland

**Keywords:** photon-counting CT, forensic radiology, postmortem imaging, virtual autopsy (VIRTOPSY), paleoradiology

## Abstract

This article explores the potential of photon-counting computed tomography (CT) in forensic medicine for a range of forensic applications. Photon-counting CT surpasses conventional CT in several key areas. It boasts superior spatial and contrast resolution, enhanced image quality at low x-ray energies, and spectral imaging capabilities that enable more precise material differentiation. These advantages translate to superior visualization of bone structures, foreign bodies, and soft tissues in postmortem examinations. The article discusses the technical principles of photon-counting CT detectors and highlights its potential applications in forensic imaging, including high-resolution virtual autopsies, pediatric forensic CT, trauma analysis, and bone density measurements. Furthermore, advancements in vascular imaging and soft tissue contrast promise to propel CT-based death investigations to an even more prominent role. The article concludes by emphasizing the immense potential of this new technology in forensic medicine and anthropology.

## Introduction

Since the early 2000s, virtual autopsy has emerged as a valuable tool in forensic medicine (1). It utilizes non-invasive radiological imaging, primarily computed tomography (CT) and magnetic resonance imaging (MRI), to examine deceased persons and potentially replace or supplement traditional autopsies in forensic death investigations (2). For this purpose, standard radiological imaging systems are utilized and the examination protocols are adapted for postmortem use (3). Forensic investigations therefore benefit from incorporating the latest advancements in technical radiology. A recent example is the use of ultra-high field 7 Tesla MRI for virtual autopsies. Due to the inherently high image resolution and higher sensitivity to the susceptibility effect at 7 Tesla compared to 3 Tesla MRI, 7 Tesla MRI can impressively illustrate the extent of cerebral tissue lesions in cerebral gunshot wounds (4). Additionally, small hemorrhages (microbleeds) could be detected more easily on 7 Tesla MRI for the same reason (5). Compared to its use in postmortem adult imaging, ultra-high-field MRI is anticipated to play an even more prominent role in postmortem pediatric imaging (6). With regard to postmortem forensic CT, micro-CT scanners are utilized to produce high-resolution datasets of skeletal structures (7). In virtual autopsies, micro-CT scanners help to examine fetuses, but these are first immersed in an iodine contrast solution for several days to improve the visibility of the soft tissue (8). In adults, the use of such high-resolution CT scanners precludes non-invasive imaging. A body part or sample has to be removed from the body in order to place it in the micro-CT scanner. While whole-body CT scanners offer submillimeter resolution, they fall short of the resolution capabilities of micro-CT scanners. In 2009, whole-body CT scanners from leading manufacturers typically employed a reconstruction matrix of 512 × 512 pixels (effects in-plane voxel size) and a slice thickness of 0.6 mm (ranging from 0.5 to 0.625 mm), defining their voxel resolution (9). Therefore, a reconstruction field-of-view (FOV) of around 300 × 300 mm, typically employed for postmortem head scans (3), yielded a near-isotropic voxel size of approximately 0.6 mm^3^. The latest advancements in CT scanner technology, including the adoption of 1,024 × 1,024 pixel matrices and thinner slices, promise considerable improvements in spatial resolution. This translates to enhanced diagnostic accuracy in virtual autopsy procedures. Furthermore, spectral CT technologies offered new possibilities for virtual autopsies. These include the use of dual-energy CT for postmortem assessment of hepatic steatosis through Rho/Z and fat fraction measurements, for material differentiation of lodged projectiles and drug packets in the body, or for reducing metal artifacts (10–13). A technological leap in CT imaging occurred in 2021 with the approval of the first photon-counting CT system for clinical use (14). This technology promises considerable improvements in image quality by offering both high resolution and the ability to capture spectral data (15). Postmortem photon-counting CT could set new standards in diagnostics not only in radiology but also in postmortem forensic imaging.

This article briefly discusses the technical principle of photon-counting detectors and the resulting advantages and extended possibilities compared to previous CT detector systems and then gives an outlook on their use in forensic postmortem imaging.

## Technical principle of photon-counting CT

Photon-counting CT uses photon-counting detectors (PCDs). Before diving into the technical principles of PCDs, it's essential to recall how conventional energy-integrating detectors (EIDs) work. EIDs detectors use a scintillator layer coupled with photodiodes at the bottom (16). When x-ray photons strike the scintillator layer, it emits visible light with an intensity proportional to the energy of the photons. However, EIDs add up the energy of all x-ray photons they detect within a specific timeframe and thus they cannot distinguish the energy of individual photons (17). In addition, the scintillator layer is divided into individual cells by optically opaque septa (thickness: approx. 0.1 mm) to prevent optical crosstalk, which creates “dead zones” in which x-ray photons cannot be detected (16). The light generated is converted into electrical signals by the photodiodes. The detector responsivity is, however, depending on the energy of the photons, which means that the signal is weighted more by photons with higher energy than by those with lower energy. Furthermore, the low-level analog electric signal from photodiodes are prone to electrical noise, especially at low x-ray flux. This results in increased image noise and instability of CT numbers at low energies.

Unlike EIDs, PCDs use a semiconductor layer to directly convert x-ray photons into electrical signals (16). When a photon hits this layer, it creates an electron-hole pair, which is then separated by a high voltage applied between a cathode and a pixelated anode (17). The electron, which moves towards the pixelated anode, induces a short current pulse which is converted into a voltage pulse. PCDs therefore “count” the individual photons, and detect their individual energies. In this way, an energy spectrum can be created that can be divided into bins. In addition, a low threshold bin can be set to exclude very low-energy events, which potentially represent electronic noise, from contributing to the CT reconstruction and thus stabilize CT numbers even at low energies. In general, semiconductor-based PCDs exhibit largely energy-independent detector responsivity, which means that photons are not weighted differently based on their energy, resulting in improved image quality at lower energies (16). This allows a higher contrast-to-noise ratio to be achieved (17). Dividing the energy spectrum into bins, known as bin imaging, also enables better separation of the energy ranges than was previously possible with kV-switching or dual-source dual-energy techniques, which are limited by slightly overlapping energy spectra. The number of possible bins is stated as two to eight (17). In addition, the photon-counting CT scanner offers 16-bit reconstructions, which means that a data set can basically contain up to 2^16^ gray values. Since PCDs do not have septa, there are inherently no “dead zones” between pixels. This allows for smaller sub-pixel sizes, potentially reaching less than 0.2 mm × 0.2 mm, which considerably enhances spatial resolution. PCD technology also poses challenges, though. For instance, “K-escape peaks”, where the energy of an x-ray photon is miscounted due to interactions with the detector material, and “charge sharing”, where electrical pulses are shared between neighboring detector cells, leading to incorrect energy counts (16).

## Photon-counting CT in forensic medicine

**Figure 1:**
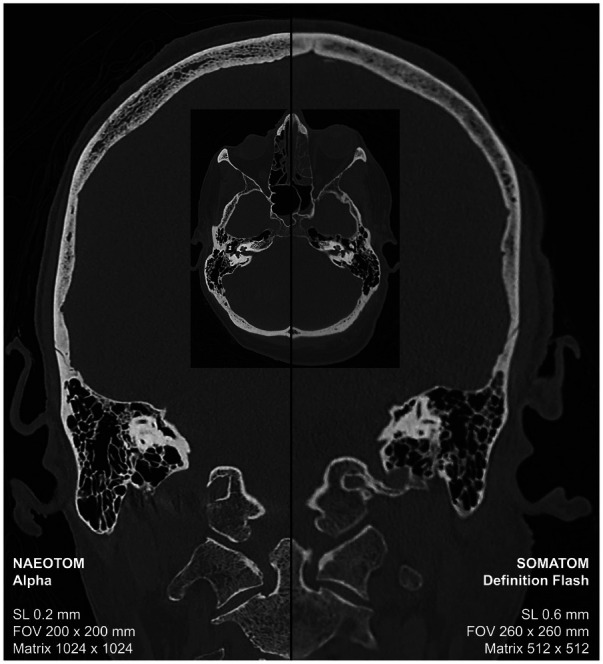
Postmortem CT scan for the virtual autopsy of a deceased person using photon-counting CT (left-sided) and conventional CT with energy-integrating detectors.

Using photon-counting CT, a voxel resolution can be achieved that raises this imaging modality from the macroscopic to the mesoscopic level. Accordingly, fine bone structures and skeletal injuries can be detected and sharply imaged in postmortem CT examinations as part of a virtual autopsy (Figure 1). In virtual autopsy, a whole body CT scan is considered the baseline scan (3). For a whole-body examination of 2 m scan length, a slice thickness of 0.2 mm (without overlapping the reconstructed slices) would result in a stack of ten thousand slices. The sheer volume of data exceeds the processing capabilities of current evaluation stations. Moreover, without the aid of artificial intelligence, it would be virtually impossible to visually inspect and accurately assess such a vast collection of images. Pediatric postmortem CT imaging, where high resolution is paramount, could significantly benefit from the implementation of high-resolution whole-body CT scans for fetuses, neonates, and infants, potentially leading to substantial diagnostic advancements. A high-resolution CT scan over a range of 50 cm would generate 2,500 slices with a thickness of 0.2 mm, representing an acceptable data volume for both post-processing applications and visual assessment. In adults such high-resolution reconstructions are rather valuable for add-on CT scans or reconstructions to visualize bony structures or skeletal injuries over a shorter anatomical range like the skull in more detail. Current CT systems face challenges in accurately depicting cranial hairline fractures, limiting the use of CT scan data for individualized head modeling and trauma analysis (18). Furthermore, conventional CT imaging can be challenging in the detection and definitive diagnosis of fractures within the intricate cartilage structures of the larynx (19). The superior resolution of photon-counting CT offers the potential for a considerable improvement in diagnostic accuracy for laryngeal evaluation in suspected strangulation cases (Supplementary Image 1). High-resolution imaging is also specifically requested for other additional examinations to the whole-body scan, such as dental scans to compare fine dental structures and implants with antemortem radiographs for radiological identification of a decomposed body or for virtual examination of other smaller foreign bodies in the deceased person (3).

If dental implants or foreign bodies are made of metal, not only resolution is important, but also a reconstruction that allows windowing into a high range of Hounsfield units (HU) (20, 21). Traditional CT scanners were limited to 12-bit reconstructions, generating 4,096 grayscale values with a range of −1,024–3,071 HU. To extend this range for visualizing high-attenuating materials like metal implants, special algorithms like Extended CT Scale were developed, primarily for image-guided radiotherapy (22). In contrast, photon-counting CT generates 16-bit reconstructions, resulting in 2^16^ grayscale values, which allows for windowing into a very high range to visualize metallic foreign bodies in detail, such as lodged projectiles (23) or dental fillings (Supplementary Image 2).

In addition to visualization, spectral photon-counting CT data may improve material differentiation. While, for example, the dual-energy index has so far proved suitable for classifying metallic projectiles into lead bullets and copper bullets, photon-counting based bin imaging may enable an extended classification. While dual-energy CT is limited by overlapping energy spectra, photon-counting CT enables bin imaging with threshold-based separation of energy ranges. Thanks to the PCDs, spectral data can already be generated for the planar overview (topogram) and thus bin imaging can be performed with topograms. This allows bone density to be measured in a similar way to dual-energy x-ray absorptiometry (DEXA) (24). While radiologists are more familiar with DEXA for bone density measurements and therefore prefer similar techniques like the topogram-based method, spectral photon-counting CT data enables more precise determination of bone volume to total volume (BV/TV), which is particularly valuable for biomechanical calculations (25, 26). This can be particularly valuable for forensic applications involving the individualized modeling and digital evaluation of biomechanical processes for trauma analysis in forensic death investigations.

**Figure 2:**
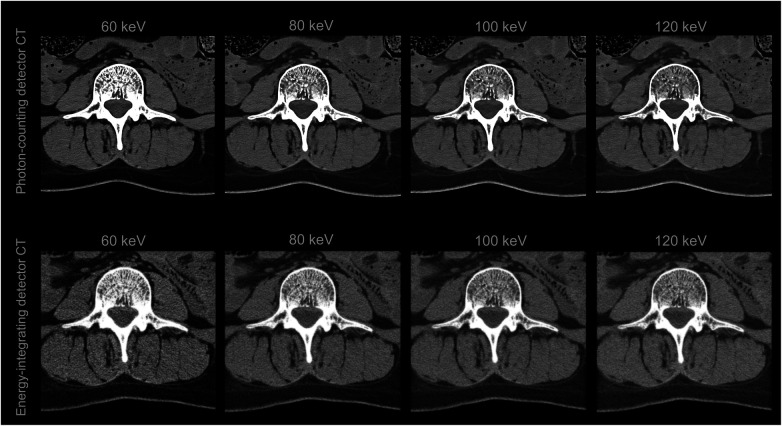
Virtual monoenergetic images (VMIs) of the third lumbar vertebra and the surrounding soft tissue of a deceased person. The VMIs in the top row are based on spectral photon-counting CT data and are of improved quality compared to those in the bottom row, which are based on conventional dual-energy CT data.

However, the standard reconstruction of raw data from photon counting CT is currently carried out neither in bin images nor in conventional polychromatic images, e.g., 120 kVp images, but in virtual monoenergetic images (VMI) (27). While VMIs were already available with dual-energy CT, photon-counting CT technology and its energy-independent detector responsivity enable enhanced VMI quality (Figure 2), particularly at lower energies (28). While low energy (low keV) VMIs enhance the visualization of soft tissue, high energy (high keV) VMIs improve the assessment of metal and other high Z_eff_ objects by reducing beam hardening artifacts (29). Advancements in VMI-based photon-counting CT hold promise for improving the accuracy of coronary artery calcium scoring, a technique gaining traction in forensic investigations due to its potential to identify cardiovascular disease, still the leading global cause of death (30). With regard to vascular stents, PCDs also demonstrate superior in-stent lumen visualization compared to EIDs (31). Postmortem angiography can benefit from the high spatial and spectral resolution of photon counting CT and new contrast agent mixtures may be used to fill the vascular system postmortem. Finally, 3D visualization with cinematic rendering also displays angiography findings in higher spatial and contrast resolution using photon-counting CT compared to energy-integrating detector CT (32). In general, the enhanced 3D visualization based on photon-counting CT data may not only improve the diagnosis of findings but also promote the learning of anatomy (Supplementary Image 3). These advancements in visualizing vascular occlusions could propel CT-based death investigations to an even more prominent role in the future.

Advanced photon-counting CT scanners using energy-independent detector responsivity have the potential to considerably enhance soft tissue contrast trough low-energy CT examinations. This is particularly advantageous for postmortem imaging, where patient radiation safety is no longer a concern, and high-dose CT scanning can be performed. By utilizing a higher tube current, the number of electrons that hit the anode in the x-ray tube is increased, producing a greater number of x-ray photons, which further improves the signal-to-noise ratio (33). While CT operators cannot directly adjust the tube current (mA), they can control the effective mA times seconds (mAs_eff_), the rotation time, and the pitch, which are interdependent (34):

$$\mathrm{mA}s_{\mathrm{eff}}=\frac{\mathrm{mA}\cdot \mathrm{rotation}\;\mathrm{time}}{\mathrm{pitch}}$$

While selecting a slower rotation time and lower pitch on the CT scanner console does not increase the mAs_eff_ value and thus the dose (CTDI_vol_), it reduces the mA value, which extends the scan time but diminishes the anode load (34). Since selecting a slow rotation time and low pitch diminishes the anode load, the CT operator can, in turn, set a higher maximum mAs_eff_, enabling high-dose CT scanning. Postmortem high-dose photon-counting CT scanning with low energy will thus optimize image quality by reducing noise and improving soft tissue contrast. However, the anatomical regions for which this method of postmortem CT imaging offers diagnostic advantages still need to be evaluated.

For a standard forensic postmortem photon-counting CT protocol, a high tube voltage (120 or 140 kVp) and a high mAs_eff_ value (300–700 mAs_eff_) are recommended, for which a low pitch (0.35) and a slow rotation time (0.5–1 s) must be set. A collimation of 120 × 0.2 mm is recommended for the whole-body scan. Although a slice thickness of 1–2 mm is suitable for the reconstruction of the entire body, a 120 × 0.2 mm collimation allows further high-resolution reconstructions of relevant anatomic regions with a slice thickness of 0.2 mm at any later time (as long as the raw data are preserved). However, a different collimation may be desired for the generation of spectral data. A second scan with a collimation of 144 × 0.4 mm is suggested. A matrix of 1,024 × 1,024 is recommended for all reconstructions.

Now that some of the potential advantages of the new photon-counting CT scanner have been presented, it can be predicted that future postmortem photon-counting CT studies hold immense potential for forensic medicine and anthropology, and it will therefore be fascinating to see what insights these forensic radiological studies will bring.

## Data Availability

The datasets presented in this article are not readily available because this article presents individual cross-sectional images from postmortem CT examinations of the head and neck area that do not allow a person to be identified. If the entire data set were made available, 3D reconstructions could make the face visible and anonymization would no longer be guaranteed. Requests to access the datasets should be directed to dominic.gascho@irm.uzh.ch.
