# Supplementary figures and images for: Photon-counting CT for forensic death investigations—a glance into the future of virtual autopsy
Source: Front Radiol. 2024 Sep 16;4:1463236. doi: 10.3389/fradi.2024.1463236 (PMC11440199; doi:10.3389/fradi.2024.1463236)

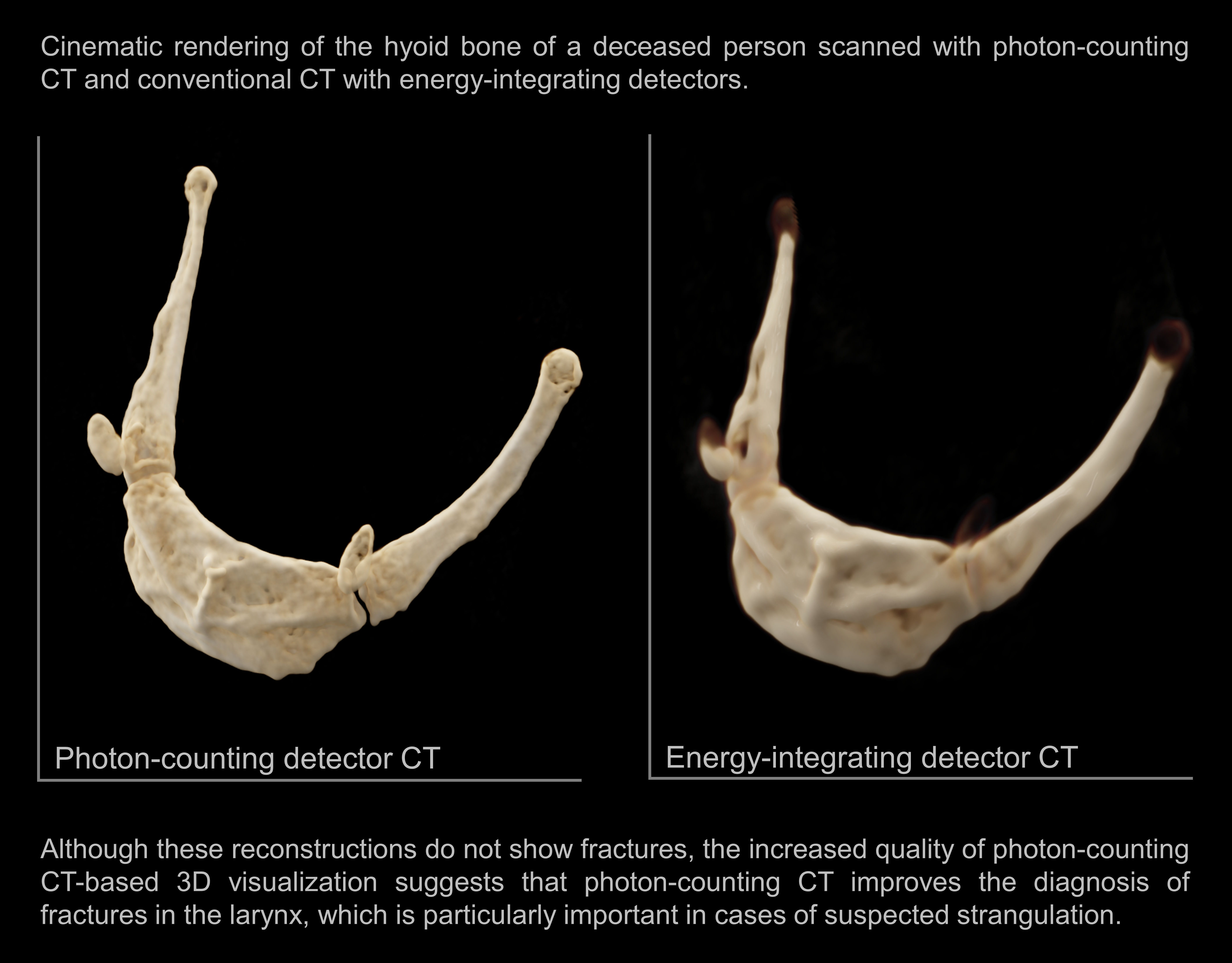

Supplement: Supplementary file 1 [file Image1.tiff]

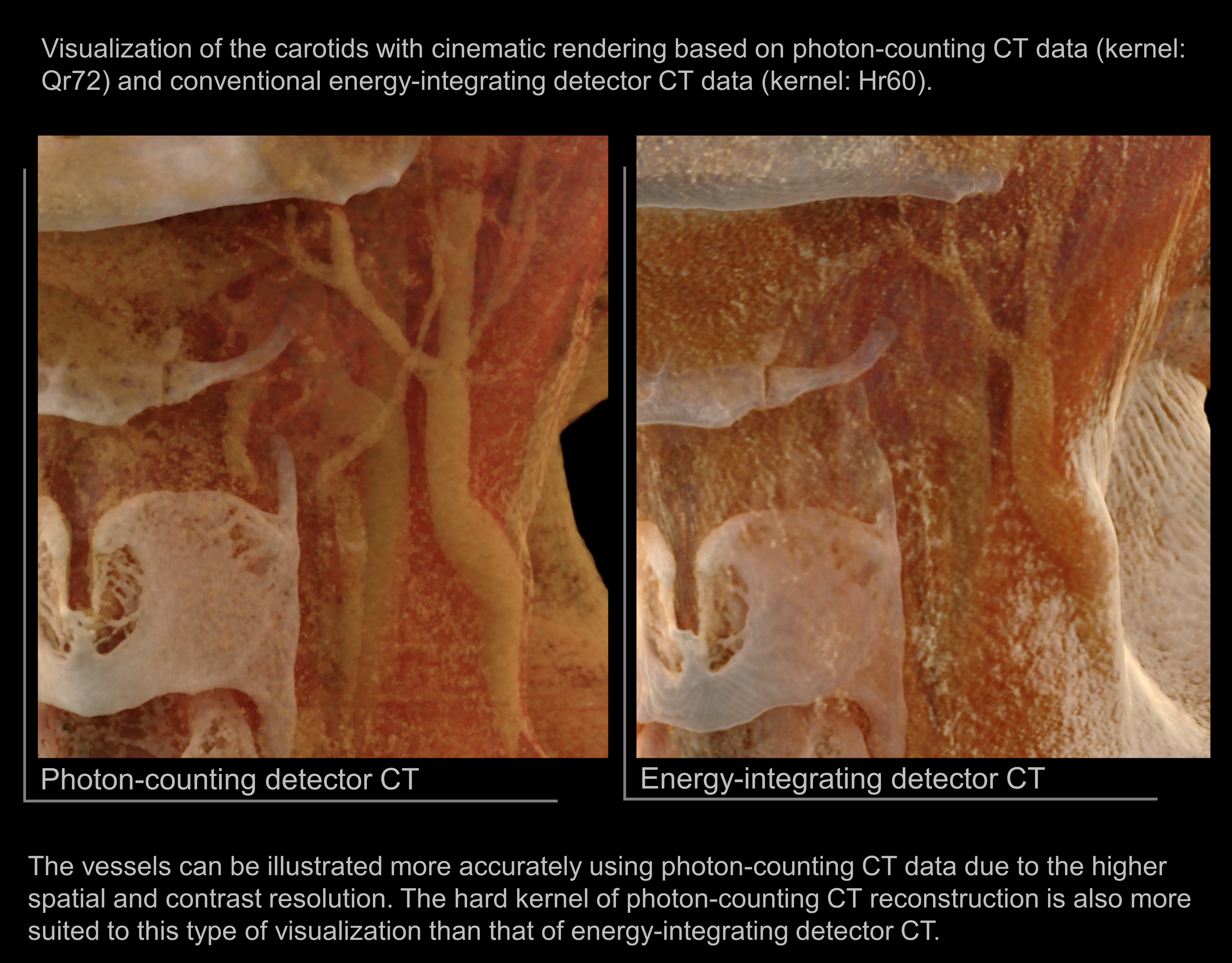

Supplement: Supplementary file 3 [file Image3.tiff]
